# An improved deep learning approach and its applications on colonic polyp images detection

**DOI:** 10.1186/s12880-020-00482-3

**Published:** 2020-07-22

**Authors:** Wei Wang, Jinge Tian, Chengwen Zhang, Yanhong Luo, Xin Wang, Ji Li

**Affiliations:** 1 School of Computer and Communication Engineering, Changsha University of Science and Technology, Changsha, 410114 China; 2 Hunan Children’s Hospital, Changsha, 410000 China

**Keywords:** Colonic polyps, Deep learning, Convolutional neural networks, Global average pooling

## Abstract

**Background:**

Colonic polyps are more likely to be cancerous, especially those with large diameter, large number and atypical hyperplasia. If colonic polyps cannot be treated in early stage, they are likely to develop into colon cancer. Colonoscopy is easily limited by the operator’s experience, and factors such as inexperience and visual fatigue will directly affect the accuracy of diagnosis. Cooperating with Hunan children’s hospital, we proposed and improved a deep learning approach with global average pooling (GAP) in colonoscopy for assisted diagnosis. Our approach for assisted diagnosis in colonoscopy can prompt endoscopists to pay attention to polyps that may be ignored in real time, improve the detection rate, reduce missed diagnosis, and improve the efficiency of medical diagnosis.

**Methods:**

We selected colonoscopy images from the gastrointestinal endoscopy room of Hunan children’s hospital to form the colonic polyp datasets. And we applied the image classification method based on Deep Learning to the classification of Colonic Polyps. The classic networks we used are VGGNets and ResNets. By using global average pooling, we proposed the improved approaches: VGGNets-GAP and ResNets-GAP.

**Results:**

The accuracies of all models in datasets exceed 98%. The TPR and TNR are above 96 and 98% respectively. In addition, VGGNets-GAP networks not only have high classification accuracies, but also have much fewer parameters than those of VGGNets.

**Conclusions:**

The experimental results show that the proposed approach has good effect on the automatic detection of colonic polyps. The innovations of our method are in two aspects: (1) the detection accuracy of colonic polyps has been improved. (2) our approach reduces the memory consumption and makes the model lightweight. Compared with the original VGG networks, the parameters of our VGG19-GAP networks are greatly reduced.

## Background

Colonic polyp is a common disease in children, which seriously affects the normal growth and development of children. Colonic polyps are more likely to be cancerous, especially those with large diameter, large number and atypical hyperplasia. If colonic polyps cannot be treated at an early stage, they are prone to develop into colon cancer. Therefore, early detection and treatment can effectively reduce the incidence of colon cancer, which is of great significance to patients [1].

Computer-aided diagnosis (CAD) of colonoscopy has been a hot spot in artificial intelligence research [2–5]. In the aspect of polyp detection CAD, in 2003, wavelet transform was used as an image classifier in the preliminary study of detecting polyps in white light colonoscopy images [6, 7]. After that, there were more CAD applications based on specific databases [8–11], but the number of images in these databases was limited, mostly less than 20. In 2015, Tyler Berzin’s team put forward the product idea of AI assisted endoscopy diagnosis [12].

In computer-aided polyp detection, feature extraction is especially important. At present, the feature extraction methods for polyp detection mainly include hand-crafted, end-to-end learning and hybrid approaches [13]. The hand-crafted feature approaches mainly use low-level image processing methods to obtain candidate boundaries of polyps, and then define the special boundary feature of each polyp with this information. Zhu et al. [14] analyzed the curvature of the detected boundary. Kang et al. [15] searched for oval shapes related to polyps. Hwang et al. [16] combined curvature analysis and shape fitting in the above two strategies. In end-to-end approaches, texture and color information are used as descriptors. Gross et al. [17] used local binary patterns (LBP) features. Ribeiro et al. [18] used deep learning approach to assist the detection of polyps. After that, there were hybrid approaches of combining hand-crafted and end-to-end learning. Tajbakhsh et al. [11] combined edge detection and feature extraction to improve the accuracy of detection. The above approaches usually use low-level simple features to detect polyps. The acquisition of these polyp features mainly extracts information such as the boundary (shape), texture, intensity, color and spatiotemporal features through artificial design program. However, only the complete and accurate extraction of colonoscopy image information can reduce the omission of polyp characteristics, which is difficult to ensure the high accuracy of polyp intelligent recognition [13].

Due to the limitation of computer algorithm and ability, the computer-aided diagnosis of colonoscopy has been limited to the basic research in the field of engineering for a long time. With the emergence of deep learning algorithm and the significant improvement of computer’s computing ability, the CAD of colonoscopy is becoming a reality [19].

In recent years, deep learning (DL) approaches, such as Convolutional Neural Networks (CNNs) [20], have achieved great success in image recognition [21], image segmentation [22], language understanding and other fields. Esteva et al. [23] used CNN model to classify skin cancer images, and the overall accuracy was higher than that of dermatologists. Gulshan et al. [24] established deep learning algorithm to recognize diabetic retinopathy, and its sensitivity and specificity both exceeded 87%. The convolution neural network algorithm was established to determine the depth of gastric cancer infiltration, and its abstract recognition ability was better than that of endoscopists [25]. Park et al. [26] used CNN to automatically extract diagnostic features from colonoscopy images. Tajbakhsh et al. [27] proposed a new polyp detection method based on convolutional neural network, enabling more accurate polyp localization. Urban et al. [28] applied CNN system to colonoscopy images, and the CNN identified polyps with a cross-validation accuracy of 96.4%.

Colonoscopy is easily limited by the operator’s experience, and factors such as inexperience limitation and visual fatigue will directly affect the accuracy of diagnosis. The CAD application of DL in colonoscopy can effectively simplify complicated diagnosis steps and improve the efficiency of medical diagnosis. The DL-based CAD can prompt endoscopists to pay attention to polyps that may be ignored in real time, improve the detection rate, and reduce missed diagnosis. In addition, the results of CAD can be used to expand the dataset so that we can conduct a convenient review of the patient’s colonoscopy images and further train the network to improve the classification performance. Based on this, we cooperate with Hunan children’s hospital to carry out the research on the assisted diagnosis of colonic polyps in children, so as to improve the detection level of intestinal polyps in children.

## Methods

Different from the traditional detection method, we apply the image classification method of DL network to the colonic polyp detection. Due to the variety of colonic polyps in morphology and the complexity of intestinal environment, it is necessary to choose the CNN model with a high degree of non-linear. The classical VGGNets and ResNets show excellent recognition ability on the labeled colonic polyp datasets. Based on the above networks, we propose two new network structures by using global average pooling, which are VGGNets-GAP and ResNets-GAP. The improved networks have outstanding classification performance, and also greatly reduce the number of parameters compared with the original networks.

**Fig. 1:**
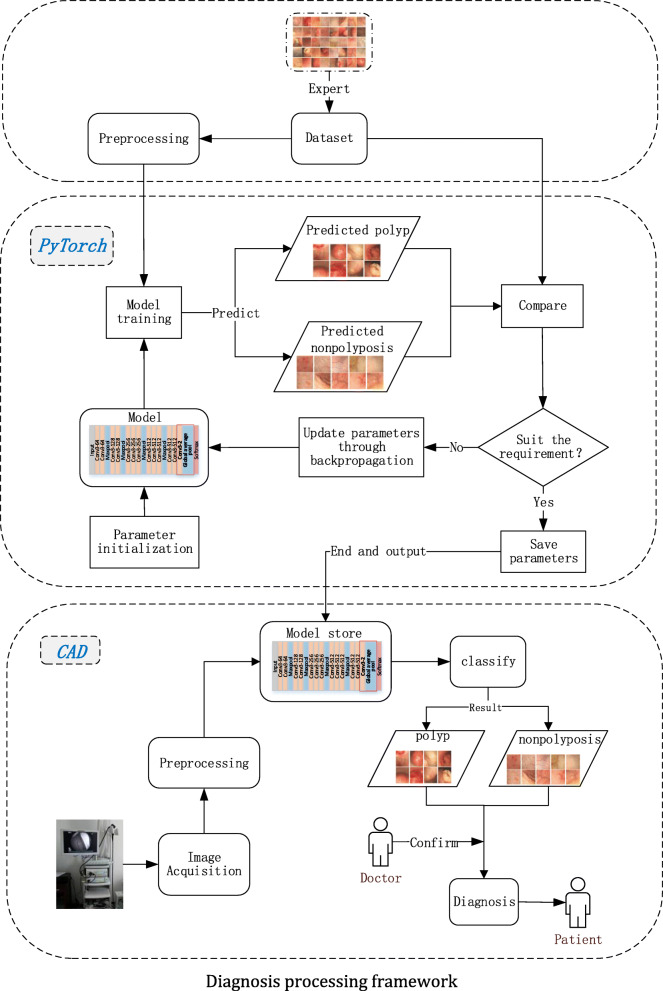
Diagnosis processing framework

In the task of colonic polyp recognition based on DL network, the medical images are directly fed into the trained network model, and the diagnosis results are given by the system, which can give doctors a clear prompt. Doctors can judge more carefully according to the results. Such assistance can effectively help endoscopists reduce missed diagnosis and improve the accuracy of colonic polyp diagnosis. The processing framework is shown in Fig. 1.

### Datasets

**Fig. 2:**
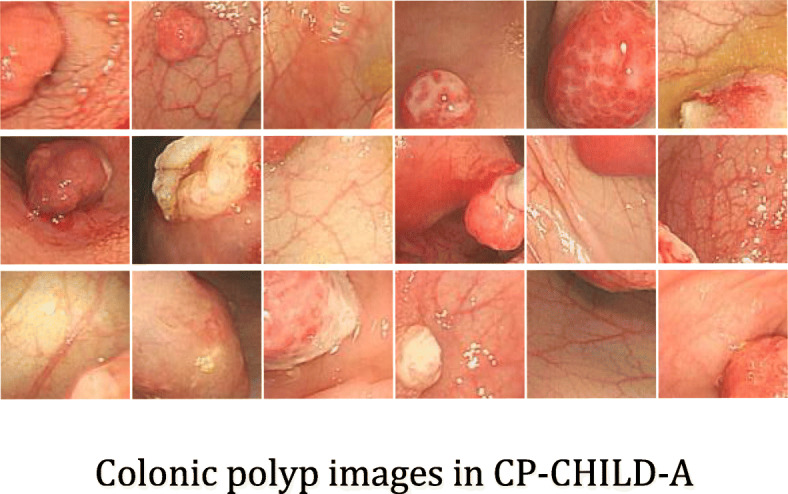
Colonic polyp images in CP-CHILD-A

**Fig. 3:**
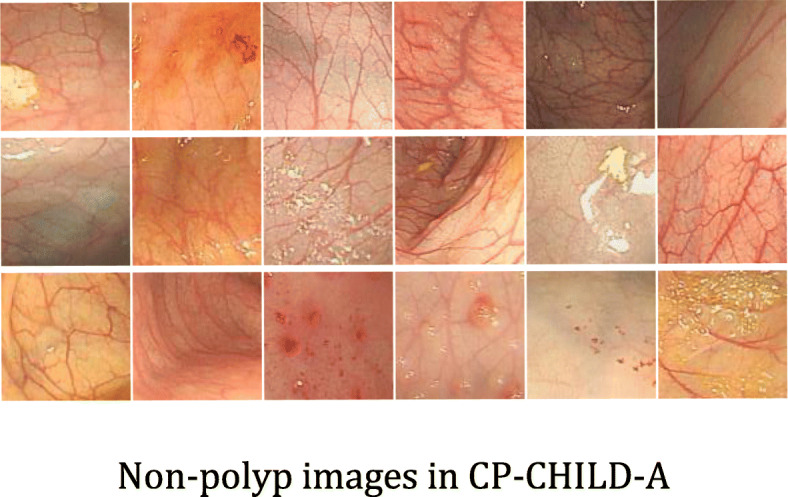
Non-polyp images in CP-CHILD-A

At present, there are very few public datasets related to colonic polyps and few images in the available datasets of colonic polyps. Compared with adults, children’s colonic polyps are easier to treat. Therefore, cooperating with Hunan children’s hospital, we collected colonoscopy images of 1600 children from Hunan children’s hospital. The age range of the patients is from 0 to 18 years old, and the average age is 3.5 years old. With the patient’s knowledge, we used these data to label two colonic polyp datasets, i.e., CP-CHILD-A dataset and CP-CHILD-B dataset. We selected 10,000 colonoscopy RGB images taken by Olympus PCF-H290DI from March 2018 to April 2019 for further processing. There are black edges around the pictures caused by the device, the pixel values of these black edges are close to 0, which are useless information features. So we first cut them before labeling, and unified the images’ size to 256 × 256. The unified images were then handed over to 4 endoscopists of Hunan children’s hospital. The endoscopists compared pathology to determine whether the image should be classified, and deleted some blurred and basically completely immersed in intestinal fluid, completely covered by feces or food debris. After labeling, we get the CP-CHILD-A dataset, which contains 1000 colonic polyp images, and 7000 normal or other pathological images, as shown in Figs. 2 and 3, respectively.

Colonic polyp detection is difficult for the following reasons: (1) Other colonic lesions, such as inflammatory bowel disease, ulcerative colitis pictures, etc., may cause bleeding, follicles, etc. their pictures look like polyp pictures. (2) Many of the polyps in the picture do not appear in the field completely, and some of them even only appear in the corner of the picture. (3) Light and shooting angle also affect the imaging quality.

The method we used to divide the training set and test set is the hold-out method. We randomly selected 6200 images from 7000 non-polyp images and 800 images from 1000 colonic polyp images to form a training set. The remaining 800 non-polyp images and 200 polyp images are used as test set. Because data enhancement is used in training and testing, including random horizontal rotation, random vertical rotation, random rotation of a certain angle between + 90° and − 90°, brightness and contrast change, which greatly increase the amount of data. Therefore, the total number of image samples in the experiments is 40,000, which is 5 times of the original data.

**Fig. 4:**
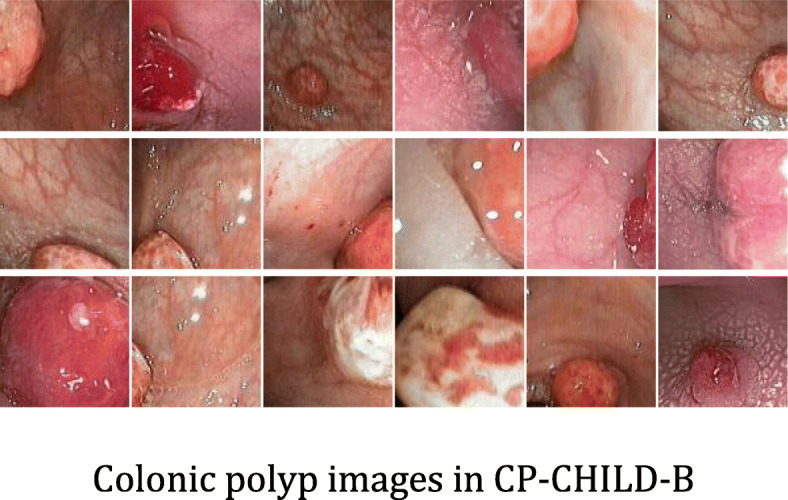
Colonic polyp images in CP-CHILD-B

**Fig. 5:**
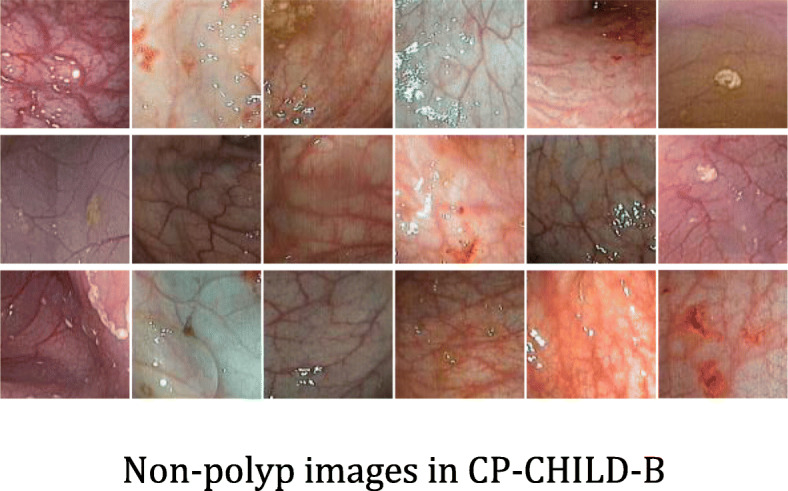
Non-polyp images in CP-CHILD-B

In order to verify the generalization ability of the models, we also selected the colonic images taken by FUJIFLIM EC-530wm to form the CP-CHILD-B dataset. After the same processing as the images in CP-CHILD-A dataset, we obtained the small CP-CHILD-B dataset containing 1500 images, which consist of 400 colonic polyp images, 1100 normal or other pathological images. The training set includes 800 non-polyp images, 300 polyp images, and the test set consists of 300 non-polyp images and 100 polyp images. The polyp and non-polyp images are shown in Figs. 4 and 5. Compared with CP-CHILD-A, the images in CP-CHILD-B are darker and there is a large difference in color between the images.

### VGGNets

VGGNet is a deep convolutional neural network model developed by Simonyan and Zisserman [29]. It explores the relationship between the depth of convolutional neural network and its performance. By repeatedly stacking 3 × 3 small convolution kernel and 2 × 2 maximum pooling layer, the convolutional neural networks with 16 ~ 19 layers of depth are successfully constructed.

In VGGNets, the parameters are mainly concentrated in the last three fully connected layers, so we can improve the performance by deepening the network. In particular, when polyps appear in the endoscopic field of vision, many of them are not complete and have no clear edges. Some polyps are covered by plica, immersed in intestinal fluid, covered by feces or food debris, and out of the camera’s view, etc. So it is exceedingly difficult to extract the edge information and texture features of the target by using the large convolution kernel, while the 3 × 3 convolution kernel can extract the details more effectively.

**Fig. 6:**
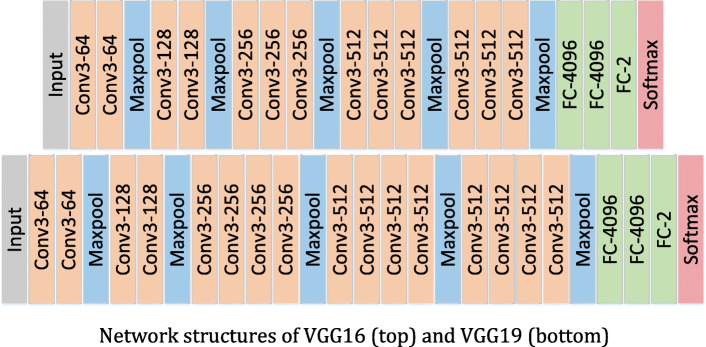
Network structures of VGG16 (top) and VGG19 (bottom)

The VGGNets used in our experiments are VGG16 and VGG19, with 16 and 19 layers respectively, as shown in Fig. 6. Each VGGNet is divided into five segments. In each segment, several 3 × 3 convolution kernels are connected in series. There is a maximum pooling layer following each segment of convolution, and 3 fully connected (FC) layers and a softmax layer are added at last. In the networks, all hidden layers use ReLU as the activation function. For simplicity, the activation function is not shown in the Fig. 6.

### ResNets

Deeper networks often have better feature extraction ability and performance. But with the increase of network depth, the degradation problem will appear, i.e., with the increase of training times, the accuracy will reach saturation or even decline. The residual network model proposed by He et al. [30] effectively solves this problem.

**Fig. 7:**
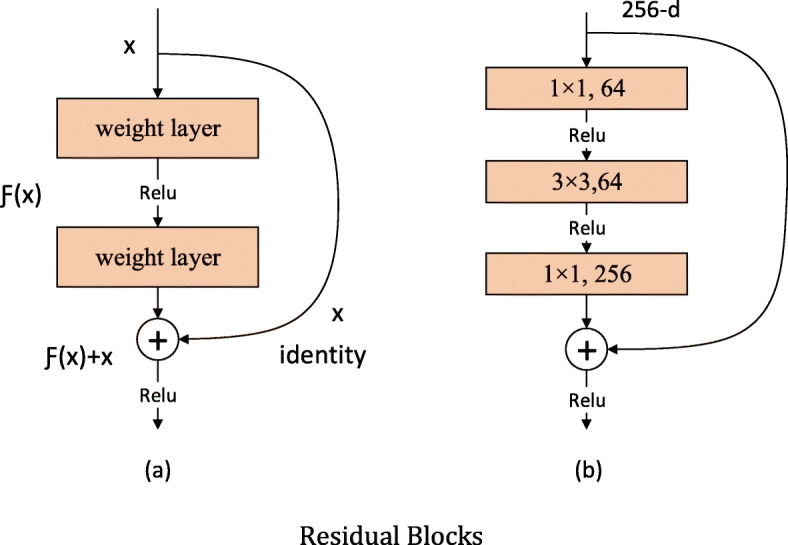
Residual Blocks

In ResNets, identity mapping is applied to optimize the residual part in order to better highlight the changes, as shown in Fig. 7a. Compared with the general convolution, the residual block uses skip connection to realize identity mapping and make it bypass the nonlinear transformation, as the curved line in Fig. 7. ResNets are stacked by residual blocks, so the network is easier to optimize and its depth can be greatly increased, both of which can improve the recognition accuracy [31].

**Fig. 8:**
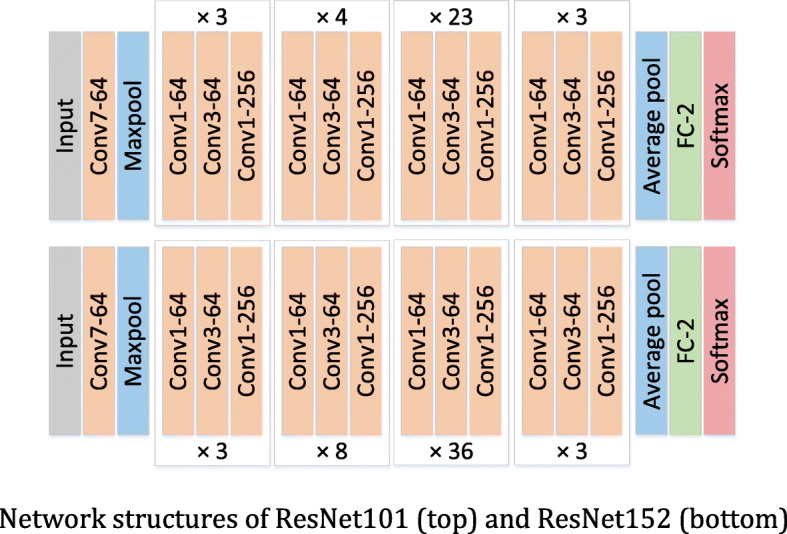
Network structures of ResNet101 (top) and ResNet152 (bottom)

The addition skip connection of the identity mapping can effectively increase the training speed of the model and improve the training effect. The residual networks used in our experiments are ResNet101 and ResNet152. The residual blocks used in these two ResNets are shown in Fig. 7b, which greatly reduce the number of parameters. The concrete network structures of ResNet101 and ResNet152 are shown in Fig. 8.

### Net-GAP

For image classification, the feature maps generated by the last convolutional layer in CNN are usually fed into a fully connected layer, and finally connected with soft-max logistic regression layer [32]. However, the fully connected layer not only brings a huge number of parameters, but also makes the network easily fall into over-fitting, which leads to the weak generalization ability of the network. Lin et al. [33] proposed the global average pooling (GAP) method for the first time. Different from the traditional FC layer, GAP layer applies global average pooling to the whole feature map, so that each feature map only gets one output. GAP greatly reduces the number of parameters, thus greatly simplifying the network and avoiding over fitting. By using GAP, each feature map has only one output feature. This one-to-one correspondence mode of feature map and category strengthens the relationship between the credibility of feature map and concept (category), and makes the classification task highly understandable.

**Fig. 9:**
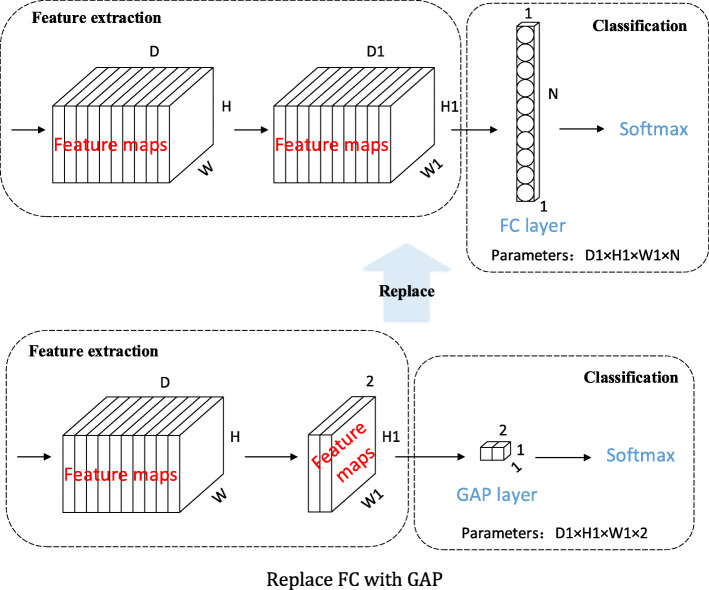
Replace FC with GAP

Based on the above idea, we combine VGG16, VGG19, ResNet101 and ResNet152 with GAP in this paper. By replacing the FC layers with GAP, four new network structures, named Net-GAP, where the “Net” is the original neural networks’ model before replacement, are obtained. The rule of replacement is shown in Fig. 9.

**Fig. 10:**
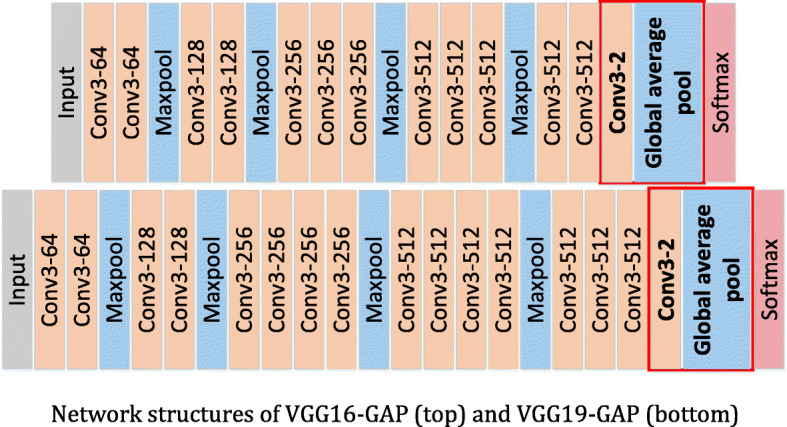
Network structures of VGG16-GAP (top) and VGG19-GAP (bottom)

**Fig. 11:**
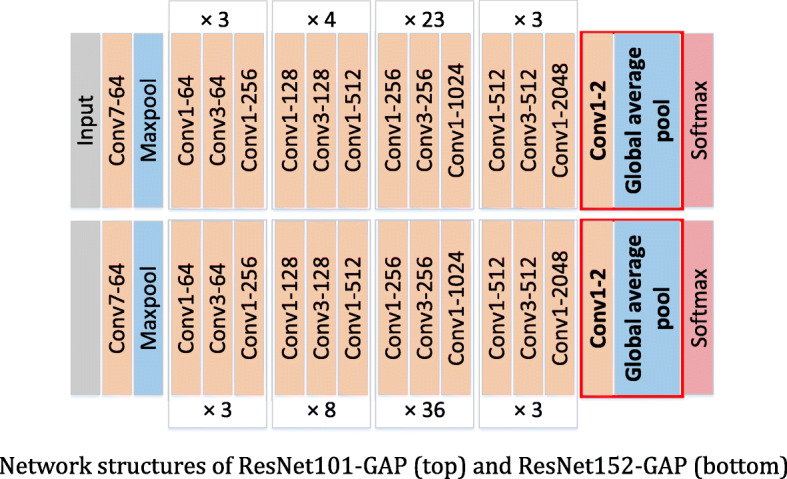
Network structures of ResNet101-GAP (top) and ResNet152-GAP (bottom)

The structures of VGGNets-GAP and ResNets-GAP are shown in Figs. 10 and 11 respectively, in which the red boxes are used to identify the changes relative to the original networks.

After the network structure is improved, the number of parameters is changed. The numbers of parameters of the VGGNets and VGGNets-GAP are shown in Table 1. By using GAP, the number of parameters of VGGNets-GAP is reduced about 153 million compared with the original network based on the same depth. The parameters of the ResNets and ResNets-GAP are shown in Table 2. Compared with the same depth of ResNets, the parameters of ResNets-GAP are reduced about 12,300. Tables 1 and 2 show that the GAP-based networks can effectively reduce the complex of networks.

**Table 1:** Parameters number of VGGNets and VGGNets-GAP

Network	Number of parameters (in million)
VGG16	165.73
**VGG16-GAP**	**12.37**
VGG19	171.05
**VGG19-GAP**	**17.68**

**Table 2:** Parameters number of ResNets and ResNets-GAP

Network	Number of parameters (in thousand)
ResNet101	42,516.546
ResNet101-A	42,504.260
ResNet152	58,160.194
ResNet152-GAP	58,147.908

### Implementation details

In our experiments, PyTorch is chosen as the deep learning framework. In order to better analyze and compare the detection results of different network models, all experiments are carried out on the same platform and environment. The experimental platform configuration is shown in Table 3.

**Table 3:** Experimental environment configuration

Configuration	Configuration parameter
operating system	Windows 10
CPU	Intel i7 3.30GHz
GPU	GTX1080Ti(11G)
RAM	16G/DDR3/2.10GHz
cuDNN	CuDNN 10.0
CUDA	CUDA10.0
Frame	PyTorch

The pre-training model on ImageNet [29] is used to initialize the parameters. In this paper, the stochastic gradient descent (SGD) method is adopted to minimize the loss function, that is, one batch of data is used instead of all data for gradient operation. The batch size of training set is set to 16, and that of test set is set to 4. In the process of training, the method of learning rate decay is introduced, that is, with the increase of the iterations, the learning rate decreases gradually. Learning rate decay can ensure that the model does not fluctuate greatly in the later period of training, so the result is closer to the optimal solution. In the experiments, the initial learning rate of ResNets and VGGNets is set to 0.01 and 0.1, respectively. Every network trains 200 epochs in total, and the learning rate decreases to half of the previous in the 50th epoch and then decays by half every 20 epochs. The average recognition accuracy of the last 100 epochs is taken as the final accuracy, and the average values of sensitivity and specificity of the last 10 epochs are taken as the final sensitivity and specificity.

### Evaluation criteria

As the evaluation criteria used by most medical image classification models, we use accuracy, sensitivity and specificity as the evaluation criteria.

In the experiments, we set the polyp samples which are few but especially important as positive samples, and non-polyp samples as negative samples. If TP represents the number of samples belonging to polyps that are correctly classified, FP represents the number of samples belonging to non-polyp samples but falsely classified to polyps, FN represents the number of samples belonging to polyps but falsely classified, and TN represents the number of samples belonging to non-poly samples that correctly classified.

Accuracy refers to the ratio of all correct classification results in the classification model to the total input pictures. It is defined as:

1 $$ \mathrm{Accuracy}=\frac{\mathrm{TP}+\mathrm{TN}}{\mathrm{TP}+\mathrm{TN}+\mathrm{FP}+\mathrm{FN}} $$

Sensitivity, also known as Recall, is the proportion that the model predicts the polyps correctly to all the results of the polyp samples, which is defined as:

2 $$ \mathrm{TPR}=\frac{\mathrm{TP}}{\mathrm{TP}+\mathrm{FN}} $$

Specificity is the proportion that the model predicts the non-polyps correctly to all the results of the negative samples:

3 $$ \mathrm{TNR}=\frac{\mathrm{TN}}{\mathrm{TN}+\mathrm{FP}} $$

## Results

**Fig. 12:**
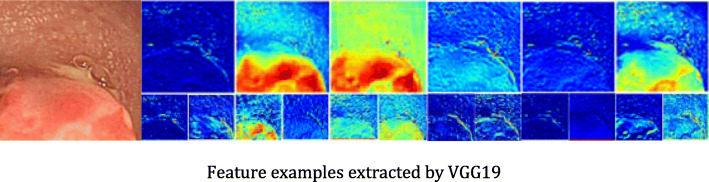
Feature examples extracted by VGG19

The close connection between the CNN layers and the spatial information make CNN particularly suitable for image processing and understanding. It can automatically extract rich relevant features from images. Figure 12 is an example of feature maps extracted by VGG19 model. The left is the original image, the upper row shows the features extracted from the second layer, and lower row shows the features extracted from the 4th layer. From Fig. 12, it can be found that most texture and detail features are extracted by the shallow layers, while the contour and shape features are extracted by the deeper layers. Relatively speaking, the deeper the layer is, the more representative the extracted features are, but the resolution of the feature maps become lower.

**Fig. 13:**
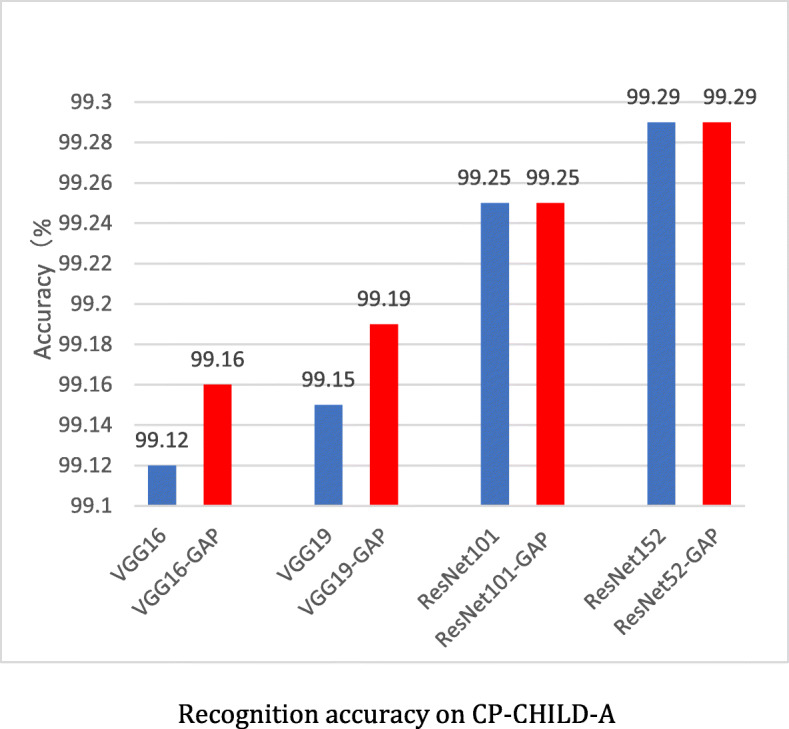
Recognition accuracy on CP-CHILD-A

We used the network models introduced in “Method” to conduct experiments on CP-CHILD-A. The average accuracies of 10 repeated experiments are shown in Fig. 13. All the 8 models perform well and the accuracies are over 99%. Our two VGGNets-GAP models not only perform slightly better than VGGNets with the same depth, but also reduce the number of parameters by about 153 million. With same depth, ResNets-GAP and ResNets have similar recognition Accuracies, but the parameters of ResNets-GAP are slightly less than that of the ResNets. Among them, ResNet152 and ResNet152-GAP have the best recognition results, and the accuracy is up to 99.29%.

The TPR and TNR of different models on CP-CHILD-A are shown in Table 4. The TPR and TNR of colonic polyp recognition based on CNNs are all over 96 and 99% respectively. Except that the TPR of VGGNets-GAP is 0.2% ~ 0.3% lower than that of VGGNets, the TPR and TNR of all other networks combined with GAP are higher than or equal to those of the original networks. Among all the networks, the TPR of ResNet152-GAP reaches 97.55% and the TNR of VGG19-GAP reaches 99.88%, which are the best scores.

**Table 4:** Recognition sensitivity and specificity on CP-CHILD-A

Model	TPR	TNR
VGG16	96.60%	99.74%
VGG16-GAP	96.40%	99.83%
VGG19	96.85%	99.81%
VGG19-GAP	96.55%	99.88%
ResNet101	96.95%	99.74%
ResNet101-GAP	97.50%	99.85%
ResNet152	97.35%	99.84%
ResNet152-GAP	97.55%	99.80%

**Fig. 14:**
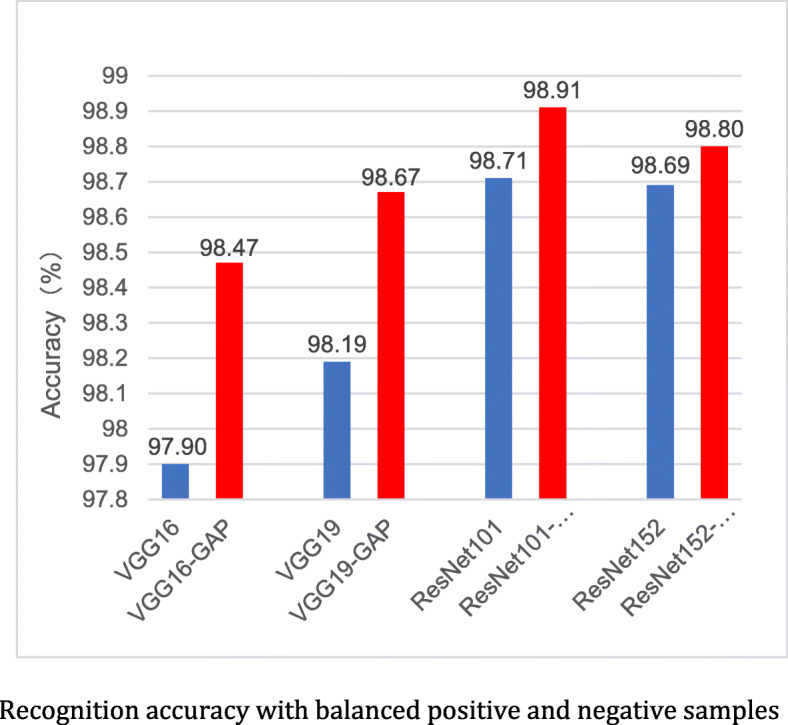
Recognition accuracy with balanced positive and negative samples

In the above experiments, the numbers of positive and negative samples in the training set are not equal. In order to further illustrate the influence of the training samples and the effectiveness of the model, we set the ratio of non-polyp images and polyp images in training set of CP-CHILD-A to 1:1. We randomly select 800 samples from 6200 non-polyp samples in the training set, and the other 5400 non-polyp samples in the training set are put into the test set. The training set of the balanced dataset contains 800 non-polyp samples and 800 polyp samples, and the test set contains 6200 non-polyp samples and 200 polyp samples. All experiments were repeated 10 times, and the average value was taken as the final experimental result. CNNs still show good performance in the test set, as shown in Fig. 14. The accuracies of the improved networks are over 98%, which are all better than the corresponding original networks. Compared with the results shown in Fig. 13, the accuracies of the proposed models are only reduced by 0.3% ~ 0.7%, which reflects the applicability of the models.

**Fig. 15:**
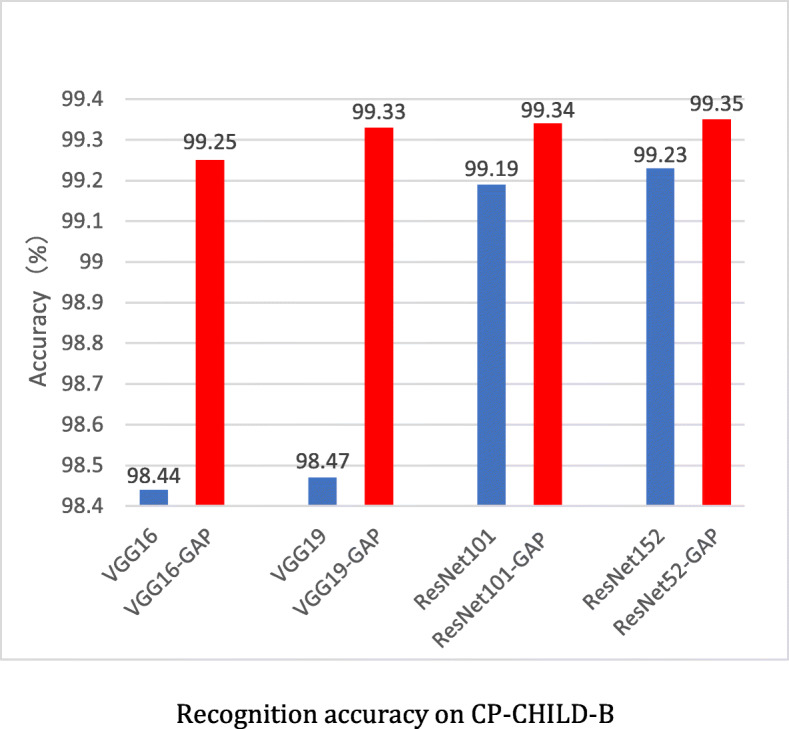
Recognition accuracy on CP-CHILD-B

To verify the generalization ability of the models, we also conducted extensive experiments on CP-CHILD-B dataset. The average accuracies of 10 repeated experiments are shown in Fig. 15. The CNNs show good performance on the test set, and the accuracies are exceeding 98%. The accuracies of the improved networks are better than those of the original networks. And the accuracies of ResNet101-GAP and ResNet152-GAP are all over 99.3%.

The sensitivity and specificity of different models on CP-CHILD-B are shown in Table 5. All the sensitivity and specificity of colonic polyp recognition based on CNNs are over 96 and 98%, respectively. Except that the TPR of ResNet152-GAP is 0.3% lower than that of ResNet152, the TPR and TNR of all other networks combined with GAP are higher than or equal to those of the original networks. Among all the models, TPRs of both ResNet101-GAP and ResNet152 reach 98%, and the TNRs of VGG16-GAP and VGG19-GAP reach 99.97%.

**Table 5:** Recognition sensitivity and specificity on CP-CHILD-B

Model	TPR	TNR
VGG16	96.50%	98.80%
VGG16-GAP	96.80%	99.97%
VGG19	96.60%	99.00%
VGG19-GAP	97.60%	99.97%
ResNet101	97.40%	99.80%
ResNet101-GAP	98.00%	99.83%
ResNet152	98.00%	99.60%
ResNet152-GAP	97.70%	99.93%

## Discussion

From the “results” section, we can see that the recognition results of 8 CNN models in the experiments have little difference between CP-CHILD-A and CP-CHILD-B. Among the four methods (with GAP) we proposed in this paper, the classification accuracies of residual structures are relatively better. Although the accuracies of VGGNets-GAP are a little less than those of the residual structures, but its number of parameters is much less than that of the original network, so it use less memory in the application. Overall, the results of VGGNets-GAP network are the best.

## Conclusions

The combination of medical image processing and neural network is a new branch and industry hotspot in the field of digital medicine, and its application in the medical field has received extensive attention. Colonoscopy is easily limited by the operator’s experience, and the factors such as inexperience and visual fatigue will directly affect the accuracy of diagnosis. Cooperating with Hunan children’s hospital, we collected colonoscopy images from the database of the gastrointestinal endoscopy room to label the colonic polyp datasets. We further proposed the application of CNNs in colonoscopy for assisted diagnosis.

In the experiments, we use the classical CNN models VGGNets and ResNets. Combining them with global average pooling, we propose the new network structures VGGNets-GAP and ResNets-GAP. The accuracies of all models in datasets exceed 98%. The TPR and TNR are above 96 and 98% respectively. The experimental results show that the proposed approach has good effect on the automatic detection of colonic polyps. In particular, the two VGGNets-GAP networks not only have high classification accuracies, but also have much fewer parameters than those of VGGNets. The reduction of model’s parameters has great benefits for reducing the memory consumption and making the model lightweight.

## Data Availability

The datasets used during the current study are available at the website 10.6084/m9.figshare.12554042.

## Abbreviations

GAP: Global average pooling
CAD: Computer-aided diagnosis
CNN: Convolutional Neural Network
DL: Deep learning
FC: Fully connected
SGD: Stochastic gradient descent
